# Characterisation of gut, lung, and upper airways microbiota in patients with non-small cell lung carcinoma: Study protocol for case-control observational trial

**DOI:** 10.1097/MD.0000000000013676

**Published:** 2018-12-14

**Authors:** Rea Bingula, Marc Filaire, Nina Radosevic-Robin, Jean-Yves Berthon, Annick Bernalier-Donadille, Marie-Paule Vasson, Emilie Thivat, Fabrice Kwiatkowski, Edith Filaire

**Affiliations:** a University of Clermont-Auvergne, UMR 1019 INRA-UCA, Human Nutrition Unit (UNH), Clermont-Ferrand; b Centre Jean Perrin, Thoracic Surgery Department, Clermont-Ferrand; c INSERM U1240, University Clermont Auvergne, Centre Jean Perrin, Department of Pathology, Clermont-Ferrand; d Greentech SA, Biopole Clermont-Limagne, Saint-Beauzire; e UMR0454 MEDIS, INRA/UCA, Saint-Genes-Champanelle; f Centre Jean Perrin, CHU Gabriel-Montpied, Clinical Nutrition Unit, Clermont-Ferrand; g University of Clermont-Auvergne, INSERM U1240 Imagerie Moléculaire et Stratégies Théranostiques, Clermont-Ferrand; h Centre Jean Perrin, Clinical Research Department, Clermont-Ferrand, France.

**Keywords:** chemotherapy, gut-lung axis, immune response, microbiota, non-small cell lung cancer, tumour microenvironment

## Abstract

**Background:**

Several studies have confirmed the important role of the gut microbiota in the regulation of immune functions and its correlation with different diseases, including cancer. While brain-gut and liver-gut axes have already been demonstrated, the existence of a lung-gut axis has been suggested more recently, with the idea that changes in the gut microbiota could affect the lung microbiota, and vice versa. Likewise, the close connection between gut microbiota and cancer of proximal sites (intestines, kidneys, liver, etc.) is already well established. However, little is known whether there is a similar relation when looking at world's number one cause of death from cancer—lung cancer.

**Objective:**

Firstly, this study aims to characterise the gut, lung, and upper airways (UAs) microbiota in patients with non-small cell lung cancer (NSCLC) treated with surgery or neoadjuvant chemotherapy plus surgery. Secondly, it aims to evaluate a chemotherapy effect on site-specific microbiota and its influence on immune profile. To our knowledge, this is the 1st study that will analyse multi-site microbiota in NSCLC patients along with site-specific immune response.

**Methods:**

The study is a case-controlled observational trial. Forty NSCLC patients will be divided into 2 groups depending on their anamnesis: Pchir, patients eligible for surgery, or Pct-chir, patients eligible for neoadjuvant chemotherapy plus surgery. Composition of the UAs (saliva), gut (faeces), and lung microbiota (from broncho-alveolar lavage fluid (BALF) and 3 lung pieces: “healthy” tissue distal to tumour, peritumoural tissue and tumour itself) will be analysed in both groups. Immune properties will be evaluated on the local (evaluation of the tumour immune cell infiltrate, tumour classification and properties, immune cell phenotyping in BALF; human neutrophil protein (HNP) 1–3, β-defensin 2, and calprotectin in faeces) and systemic level (blood cytokine and immune cell profile). Short-chain fatty acids (SCFAs) (major products of bacterial fermentation with an effect on immune system) will be dosed in faecal samples. Other factors such as nutrition and smoking status will be recorded for each patient. We hypothesise that smoking status and tumour type/grade will be major factors influencing both microbiota and immune/inflammatory profile of all sampling sites. Furthermore, due to non-selectivity, the same effect is expected from chemotherapy.

## 1 Introduction

The microbiota is a consortium of different microorganisms that includes bacteria (microbiota), fungi (mycobiota), viruses, and protozoa residing on the skin and in the oral, pulmonary, urogenital and gastrointestinal (GI) cavities, with the GI tract having the highest density of microorganisms. The functional importance of the microbiota to the host is undeniable, involving functions that range from the breakdown of complex dietary polysaccharides to competing with pathogens and modulating the mucosal and immune system in general.^[1]^ Gut dysbiosis is now considered to be an underlying cause of a wide range of GI diseases and an emerging number of non-GI conditions such as obesity and cardiovascular disease, as well as a range of psychiatric diseases.^[2]^ Recently, an emerging number of studies began to address the relation between gut microbiota and the lung. This relation has been referred to as the “gut-lung axis”. The basis of this axis theory lies in the “gut-lymph” theory of Samuelson et al^[3]^ The theory says that the large numbers of macrophages and other immune cells are present in the intestinal submucosa or mesenteric lymph nodes, where the majority of translocating bacteria are also found. If not eliminated by this 1st line defence, surviving bacteria, cell wall fragments or the protein fractions of dead bacteria escape with the cytokines and chemokines produced in the gut, travel along the mesenteric lymphatic system to the cisterna chyli, and subsequently enter the circulatory system. Thereby they have access to pulmonary circulation, which may lead to the local activation of dendritic cells and macrophages as well as T cell priming and differentiation. Another way to influence the pulmonary region might be through the migration of immune cells themselves, after priming and activation at the 1st site of antigen encounter, i.e. the gut mucosa. Although this theory explains the unilateral interaction, it is reasonable to speculate that this axis works the same way when it originates in the lung mucosa and lung lymph nodes.^[4]^ Moreover, nutrition can also affect both immune response and composition of our respiratory tract microbiota.^[5]^ In mice, high-fibre diet increased protection against allergic inflammation in the lung (reduced inflammatory cell infiltration), followed by a change in the gut and, to a lesser extent, the airway microbiota.^[6]^ The study also reported an increase in blood levels of circulating short-chain fatty acids (SCFAs), one of the major products of bacterial fermentation responsible of intestinal barrier integrity and known for its anti-inflammatory properties. However, no traces were found in the lung itself. On the contrary to allergic inflammation, a lack of an appropriate stimulus during the developmental phase of an immune response, as during infection, will disable a quick and effective immune reaction. This could result in undesirable consequences such as pathogen colonisation, increased susceptibility to infection, tissue damage, possible development of cancer and increased mortality.^[7,8]^ Therefore, it is clear that there is a complex network of distinct and precise stimuli that are required for executing a correct immune response. According to the gut-lung axis theory, these stimuli can originate in the gut, explaining the observed protective effect in the lung. Taking a huge step forward, the study of Routy et al (2018)^[9]^ evaluated the role of gut microbiota in responsiveness to anticancer treatment by immune checkpoint inhibitors (ICI) (PD-1/PD-L1). They showed that non-small cell lung cancer (NSCLC) patients that received antibiotic treatment (ATB) during 2 months before therapy had significantly decreased overall and progression-free survival. Similarly, ATB treatment was a predictor of ICI resistance, independent from other prognostic markers. When faecal microbiota transfers using the stool from NSCLC patients responding or not responding to therapy were performed, inoculated germ-free (GF) mice showed the same phenomenon during ICI therapy against MCA-205 tumours. Mice receiving ICI therapy that were inoculated with a responder's stool showed delayed tumour growth and accumulation of antitumour lymphocytes in the tumour microenvironment. The stool of NSCLC patients responding to ICI therapy was found to be enriched in phylum *Firmicutes*, as well as distinct genera such as *Akkermansia*, *Ruminococcus*, *Alistipes,* etc. In further experiments with GF mice, *Akkermansia muciniphila* proved to be sufficient to restore ICI therapy responsiveness, both when inoculated alone or with the stool from non-responding NSCLC patients, rectifying the response. Likewise, it was the only species that induced reactivity from patient-derived Th1 and Tc1 in vitro, and that correlated with progression-free survival. Looking at these results, it is evident that the gut microbiota plays a crucial role in the host's homeostasis and that its fine-tuned composition counts for much more than was previously thought. However, data on this topic remain scarce but directed to a promising field of a new anti-lung cancer approach that the world population is yearning for.^[10]^ Unlike the local and systemic influence of the gut microbiota, the influence on and of the lung microbiota and its products has yet to be properly assessed, both in health and disease.^[11]^

Therefore, to help to elucidate this new and extremely interesting field, we have decided to conduct a case-control observational study in patients with NSCLC. The study will include 2 groups of patients: 1st group Pchir, with patients eligible for treatment by surgery, and 2nd group Pct-chir, with patients eligible for a combined treatment consisting of neoadjuvant platinum-based chemotherapy followed by surgery.

The objectives of this study are:

(i) to characterise the gut, lung and upper airway microbiota in these patients;
(ii) to evaluate the homogeneity/heterogeneity between different microbiota within the same subject/group of patients;
(iii) to evaluate the impact of the microbiota composition on immune and inflammatory status of the patient (evaluated in the gut, blood, lung);
(iv) to evaluate the effect of chemotherapy on the site-specific microbiota (UAs, lung, gut).

While group Pchir will have only 1 time point for sample collection, group Pct-chir will have multiple time points. The latter will enable follow-up on changes in microbiota and immune markers relative to the treatment progression.

## 2 Methods and analyses

### 2.1 Ethics approval and dissemination

This protocol has been approved by the Committee for the Protection of Persons (CPP) Sud-Est VI, Clermont-Ferrand, France, and The French National Agency for Medicines and Health Products Safety (ANSM) (study ref. 2016-A01640-51). Because of the invasiveness of the sampling techniques, the requested control group was not approved by the CPP. The study was accompanied by amendment approved by ANSM in June 2018. The current protocol is entitled “Protocol MICA V3”, and presents an up-to-date version and the version in use. This study is registered with the Clinical Trials under ID: NCT03068663. The study's official name is: Characterisation of the microbiota (gut, lung, and upper airways) in patients with non-small cell lung carcinoma: exploratory study (acronym: MICA). Written informed consent is obtained from all patients before enrolment in the study. The results are planned for presentation at conferences and publication in peer-reviewed journals in early 2019. All samples will be preserved for 15 years according to the practice of the sponsoring institution (Centre Jean Perrin). Samples will be available to other investigators if they want to perform complementary studies that consider NSCLC after additional consent obtained from patient. However, because of French regulations regarding patient information files, patients’ data will not be available.

### 2.2 Study outcomes

As its primary outcome, this study will characterise the lung and UAs microbiota in 2 groups of 20 patients with NSCLC. Group Pchir will include patients eligible for surgery without chemotherapy. Group Pct-chir will include patients eligible for surgery after platinum-based chemotherapy. The UAs microbiota will be evaluated from saliva, while lung microbiota will be evaluated from 3 lung explants: “healthy” lung tissue, tumour and peritumoural tissue; and broncho-alveolar lavage fluid (BALF) from the tumour's proximity (same lobe). Following bacterial DNA extraction, microbiota will be analysed by qPCR and 16S ribosomal rRNA gene sequencing using the Illumina MiSeq platform.

Secondary outcomes of this study are set as follows:

i) to study the effect of neoadjuvant chemotherapy on microbiota by evaluating:
  a. the variation of the proportion of the phylum *Firmicutes* (as the phylum is highly represented in all of the different types of samples considered in this study),^[4]^
  b. the variation of the proportion of the bacterial genera per phylum in different types of samples (faeces, saliva, BALF, lung tissue/peritumoural tissue/tumour),
  c. the concordance of genera between sample locations (e.g. saliva vs. BALF, healthy lung tissue vs. peritumoural tissue vs. tumour);
    by qPCR and 16S rRNA gene sequencing;
ii) to study the homogeneity/heterogeneity between lung, upper airways and gut microbiota for each and between both groups (evaluated by 16S rRNA gene sequencing and qPCR) and between different time points (group Pct-chir),
iii) to evaluate immune/inflammatory status:
  a. in the gut: by dosing β-defensin 2, human neutrophil peptides HNP1-3, calprotectin (ELISA) and SCFAs (gas liquid chromatography)
  b. in plasma: by dosing plasmatic cytokines (Luminex), C-reactive protein (CRP) (ELISA) and immune cell phenotyping (flow cytometry)
  c. in the lung: by immune cell phenotyping (flow cytometry) and characterisation of immune infiltrate in lung tumour biopsies obtained during the operation (by immunohistochemistry).

### 2.3 Patient recruitment

The study pre-considers all patients diagnosed with NSCLC and presented before the Thoracic Oncologic Committee of the Jean Perrin Centre, Clermont-Ferrand, France. Inclusion criteria are presented in Table 1. Inclusion in the study is consecutive and parallel for both groups. A written informed consent is obtained from each patient participating in the study before inclusion. Depending on their diagnosis, patients are included in one of the 2 groups: Pchir (patients eligible for surgery only), or Pct-chir (patients eligible for surgery after neoadjuvant platinum-based chemotherapy).

**Table 1:**
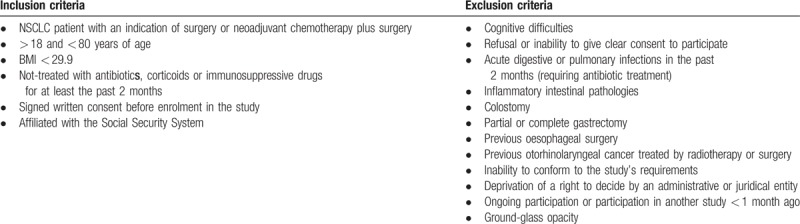
Inclusion and exclusion criteria for patients.

### 2.4 Patient and public involvement

Neither patients nor the Patient Committee were involved in the design of this study. If desired, patients can be informed of the study's results by the investigator or physician.

### 2.5 Trial design and timeline

This study is a case-control observational trial. Recruitment into the study started in May 2017 and will end in May 2019. The estimated complete duration of the study is 29 months, with an average follow-up period per patient of 1.5 and 3.5–4.5 months for the Pchir and Pct-chir groups, respectively. Intermediary analyses will take place after obtaining all samples from half of the patient quota (n = 20) regardless of the group, without interruption of the further recruitment to the study.

**Figure 1:**
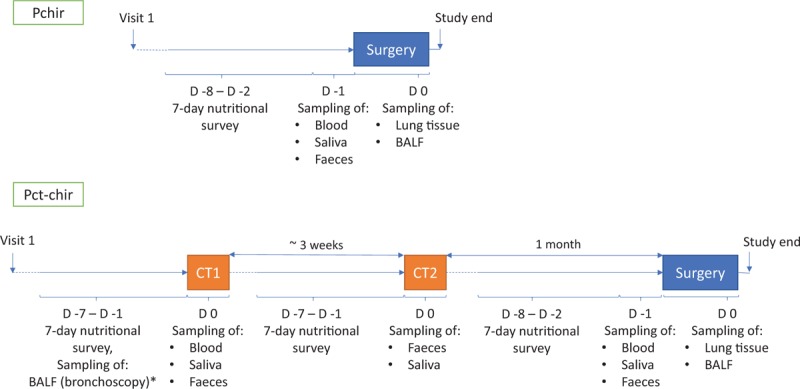
Study flowchart. BALF = broncho-alveolar lavage fluid, CT = chemotherapy, D = day, Pchir = the group of patients undergoing surgery, Pct-chir = the group of patients undergoing chemotherapy and surgery. ^∗^performed as a part of patient's standard medical care if decided by his/her physician.

The trial design is presented in Figure 1. Eligible patients meet official study personnel at an outpatient appointment (Visit 1) where all the details of the protocol are thoroughly explained. At this visit, patients give their written consent to participate in the study. Depending on their clinical diagnosis, they are assigned to one of the 2 groups (Pchir or Pct-chir). Each patient is given a protocol summary, sampling instructions and corresponding number (depending on inclusion group) of tubes for saliva, boxes for faecal samples with anaerobic atmosphere generation bags and templates for a 7-day nutritional survey. Other information is also recorded, such as smoking status and history, weight or diet modifications in the last several months/years, cohabitation or interaction with animals in their childhood/present, the environment in which patients grew up/spent their life (countryside/city), exposure to certain pollutants, e.g. related to their profession, etc. This information will serve for better understanding of the individual's inflammatory status and microbial characteristics.

As presented in Figure 1, group Pchir has sampling concentrated around 1 event—surgery. At hospital admission (D-1 before surgery), samples of saliva, blood and faeces are collected together with the nutritional survey patients made during the preceding week (D-7 to D-1). The BALF and lung tissue samples are collected during the surgery (D0) from the excised lobe.

Group Pct-chir (Fig. 1) has sampling concentrated around 3 events:

i) 1st chemotherapy cycle (CT1)—the day of CT1, samples of saliva, blood and faeces are collected together with nutritional surveys patients made during the preceding week (same as D-1 for Pchir group). After sample retrieval, patients continue with their medical care procedure. Optionally, in the days preceding the CT1 as the part of their standard medical care, patients can undergo bronchoscopy. In this case, a part of retrieved liquid (BALF) is taken for microbiota characterisation and immune cell profiling.
ii) 2nd chemotherapy cycle (CT2) —CT2 is usually 3 weeks after CT1. The day of CT2, only saliva, faeces, and nutritional surveys are retrieved. After sample retrieval, patients continue with their medical treatment.
iii) Surgery—The time elapsed between CT2 and surgery is about 1 month, depending on the patient's overall health status. The sampling for surgery is exactly the same as for group Pchir. At hospital admission (D-1 before surgery), samples of saliva, blood and faeces are collected together with nutritional surveys patients made during the preceding week (D-7 to D-1). The BALF and lung tissue samples are collected during the surgery (D0) from the excised lobe.

The strong point of this trial is that study participation does not modify the patient's standard care treatment in any way. Invasive intervention, such as sampling of the lung tissue, is done during operation on the lung already removed from the patient. The BALF sampling during operation is performed directly on the dissected lobe, posing no additional inconvenience for the patient and drastically minimising UAs contamination, which is an important advantage to this study's concept. Likewise, sampling of the BALF at CT1 for group Pct-chir is done in the scope of a standard care procedure by bronchoscopy and it is not imposed by the study. However, the latter is also an inconvenience because there will be patients from group Pct-chir who will not undergo bronchoscopy with BALF sampling since it is not prescribed by his/her physician. Another thing to consider is also the difference in sampling technique of BALF. Sampling of BALF at CT1 for group Pct-chir is done by bronchoscopy, while sampling during surgery is done directly on the excised lobe for both groups. Therefore, the 1t BALF has a higher risk of contamination by UAs, while the 2nd BALF (during operation) should have no UAs contamination and better represent luminal microbiota specific of the tumour lobe. This will be taken into account during data interpretation, and if necessary, each time point will be characterised for itself.

In conclusion, in the group Pct-chir, the effect of chemotherapy on the microbiota of saliva and faeces and immune parameters of blood and faeces will by systematically assessed. Chemotherapy's effect on immune parameters and microbiota in BALF will be assessed only if obtained data in quantity and quality will permit it (as explained above). When talking of lung tissue, “healthy” lung tissue, taken at distance from the tumour, will be considered as a control^[12]^ tissue for comparison with peritumoural and tumoural tissue, as well as with BALF. Also, characterisation will be done respective to the tumour type where possible (sufficient patient number with the same tumour type).

A demanded control group for the study was not authorised by the Ethics Committee, due to the invasiveness or evident inability to realise certain sampling steps (bronchoscopy, lung tissue sampling). Therefore, the focus will be on characterisation of the site-related microbiota and its connection to local and systemic immunity in NSCLC respective of the treatment group (Pchir or Pct-chir). Likewise, the question is whether there is an initial difference between group Pchir and group Pct-chir (before CT1), and what its nature is. Equally, how does chemotherapy modify group Pct-chir, and whether it becomes more alike or different from group Pchir regarding its different properties (microbial taxa ratios, inflammatory properties) when followed in time (from CT1 to surgery). Furthermore, obtained results considering microbiota composition and abundance, where possible, will address similar studies^[13–16]^ only in a descriptive matter and the same will be done with immune parameters.^[17–21]^

## 3 Sampling and data recording

### 3.1 Nutritional survey

The dietary habits are evaluated for each patient for the 7 days preceding chemotherapy (Pct-chir) and/or surgery (Pchir and Pct-chir). At Visit 1 (see Fig. 1), all participants receive a detailed verbal explanation, written instructions and the survey with an example. They are asked to maintain their usual dietary habits during the survey period and to record as accurately as possible the amount, type and preparation of food and fluid consumed. If they consume commercial and ready meals, they are also asked to note brand names. The quantity of food or drink can be expressed in either precise measures (weight) or in common household measures, such as cups, tablespoons, etc. In the case of any questions or ambiguities, patients are encouraged to contact the study personnel. These data will help to estimate each patient's overall nutritional status and help to explain the microbiological analysis of faecal samples following the survey, as well as the patient's immune and inflammatory status. We anticipated that the patients might change their dietary habits during the chemotherapy duration, which is why recording was requested before each chemotherapy treatment and surgery, and during 1 week. The primary objective is to use what was recorded as complementary data to the faecal microbiota analysis (sampled the day after the end of each survey), to better explain the longitudinal modification of microbiota, if any. This is due to the fact that faecal microbiota can overcome significant changes in only a few days relative to a diet change.^[22]^

### 3.2 Saliva

At Visit 1, after recording any evidence of oral health problems or injuries, each patient receives a tube for saliva collection (Sarstedt). It is necessary to fill the tube with a minimum of 1 mL of saliva (designated on the tube) on an empty stomach by the passive drooling method on the morning of hospital admission for the chemotherapy session (Pct-chir) and/or surgery (Pchir, Pct-chir) (Fig. 1). The sample is stored at −80 °C for later bacterial DNA extraction and metagenomic sequencing.

### 3.3 Blood

Blood sampling is done following the patient's admission to hospital, in the day/hours preceding the prescribed clinical treatment, depending on the group (Fig. 1). A 10 mL of fresh blood are collected in EDTA treated tubes, where 500 μL of whole blood is immediately used for immune cell phenotyping by flow cytometry. The remainder is centrifuged for 10 min 2000 × g at 4°C to obtain plasma, which is then aliquoted and stored at −80°C for further analyses (cytokine/interleukin analysis by Luminex, CRP dosage by ELISA).

### 3.4 Faeces

At Visit 1, each patient receives a sampling box, an anaerobic atmosphere generation bag (GENbag anaer, Biomérieux) and detailed printed instructions on how to handle the samples at his/her home. Faecal samples are collected following the 1-week nutritional survey (i.e. on the day of chemotherapy before the drug infusion or the day preceding surgery). In brief, sampling is done directly into the sampling box, and after removing the protective foil and placing the anaerobic atmosphere generation bag in the box, the box is closed firmly and placed in the cold (+ 4°C). Patients are asked if they have the ability to transport the sample in an insulated bag to preserve cold conditions. If there is no such possibility, the study personnel supplies the patient with the requested bag. The sampling should be done within 12 hours preceding the hospital admission and sample retrieval. Therefore, patients are asked to do the sampling the morning of hospital admission if possible. If there are any problems, the patient is asked to contact the protocol personnel to ensure that the sample is processed in time. On reception, the sample is aliquoted 3 × 1 g for bacterial DNA extraction, and 2 × 5–10 g (depending on availability) for dosage of faecal calprotectin, β-defensin 2, HNP1-3 (ELISA) and SCFAs (gas liquid chromatography). Aliquots are stored immediately at −80°C until analysis. Approximately 1 g of fresh sample is used for bacterial culture of the main functionary groups of microorganisms (total anaerobic bacteria, mucin-degrading bacteria, lactic acid producing bacteria, sulphate-reducing bacteria and *Enterobacteriaceae*).

### 3.5 Lung tissue and BALF

Only for group Pct-chir, BALF is sampled at inclusion to the study. This sample is taken as a part of patient's standard care protocol if decided by his/her physician, and is not taken as an additional sample for this study. The BAL is performed by routine bronchoscopy procedure.

Sampling of lung tissue and BALF during surgery is performed for both groups, after partial or complete pneumonectomy. The removed lung tissue is placed in a sterile vessel and the tumour position is determined by palpation. A piece of healthy lung distal to the tumour, with a minimum size of 1 cm × 1 cm × 1 cm, is then clamped. The clamp is left in place during the following procedure. The stich on the bronchus is cut away and using a sterile syringe the lung is inflated through the bronchus. Lavage is performed by instilling 2 × 40 mL of sterile physiological saline. After each instillation, the maximum amount of liquid inside the bronchus is retrieved (8–10 mL in total), poured into a sterile 50 mL tube and placed immediately on ice, designated as “BALF”. At the end, the clamped wedge is cut off and designated as “healthy lung”. A slice of the tumour, with a minimal weight of 400 mg, containing the tumour cross-section is excised along with peritumoural tissue, after which the 2 are separated based on histological difference. All tissues are frozen 1st in liquid nitrogen and then placed at −80°C for long-term storage until DNA extraction. The mirror piece of the excised tumour slice is stored in paraffin and later analysed by immunohistochemistry for characterisation of tumour infiltrate. A 3 mL of BALF are immediately used for immune cell analysis by flow cytometry and the remainder is stored at −80°C for later DNA extraction.

## 4 Methods and analyses

Saliva, “healthy” lung, peritumoural, and tumour tissue, BALF and faecal samples will be used for bacterial DNA extraction, followed by qPCR and 16S rRNA gene sequence analysis to establish microbial profiles. Fresh faeces samples will be used for bacterial culture, and frozen aliquots for dosage of faecal calprotectin, β-defensin 2, HNP1-3 (ELISA), and SCFAs (gas liquid chromatography). The BALF and plasma samples will both be analysed by flow cytometry (immune cell phenotyping), while plasma will also be used for cytokines (Luminex) and CRP (ELISA) dosage. Tumour tissue stored in paraffin will be used for the analysis of immune infiltrate by immunohistochemistry. Each procedure is explained in detail in the following sections.

### 4.1 Nutritional status

Nutritional data for each patient will be analysed using the Nutrilog 2.3 software package, a computerised database (Proform) that calculates food composition from the French standard reference.^[23]^

### 4.2 Cytokines

Stored plasma will be analysed for cytokines corresponding (but not restricted) to the following profiles: Th1, Th2, Th17, Treg. Samples will be analysed using Luminex kits: HSTCMAG-28SK, HTH17MAG-14K and TGFBMAG-64K-01 (Merck Millipore). Analyses will be conducted by the phenotyping service of CREFRE, Toulouse, France.

### 4.3 Evaluation of tumour immune infiltrate

Immunohistochemistry will be performed on tumour tissue using the specific antibodies to detect subpopulations of immune cells as follows: cytotoxic T-lymphocytes (anti-CD8, clone SP16, ThermoFisher Scientific), regulatory T-lymphocytes (anti-FoxP3, clone SP97, ThermoFisher Scientific), B-lymphocytes (anti-CD20, clone SP32, Cell Marque). The immune response checkpoint axis PD-1–PD-L1 will be assessed by anti-PD-1 (clone NAT105, Cell Marque) and anti-PD-L1 (clone 28-8, Abcam). All staining will be performed by a fully automated, standardised procedure (Benchmark XT, Ventana/Roche). The number of lymphoid cells expressing each antigen, except PD-L1, will be determined within 5 consecutive x40 microscopic fields, starting from the invasive front toward the tumour centre, used as a parameter reflecting the tumour's quantity of a given immune cell subpopulation. PD-L1 will be assessed for both immune and tumour cells and reported as the percentage of each population expressing the antigen.

### 4.4 Immune cell phenotyping

Immune cell phenotyping will be performed on fresh samples of blood (0.5 mL) and BALF (3 mL) by flow cytometry. Leukocytes will be obtained after haemolysis (solution of 155 mM NH_4_Cl, 12 mM NaHCO_3_, 0.1 mM EDTA) for 15 min at room temperature, followed by 10 min centrifugation at 600 × g. Before centrifugation, BALF will be filtered through a porous gauze to eliminate mucus and reduce the viscosity. Lymphocyte subpopulations will be phenotyped using the following antibodies: anti-CD3-VioBlue, anti-CD4-APC-Vio770, anti-CD25-APC, anti-CD127-VioBright FITC, anti-CD183 (CXCR3)-PE-Vio770, anti-CD294 (CRTH2)-PE, anti-CD196 (CCR6)-PE-Vio615, anti-CD15-FITC, anti-CD62L-PE, anti-CD11b-PE-Vio770 and Viobility 405/520 fixable dye, all purchased from Miltenyi Biotec. A T lymphocytes CD4^+^ will be characterised as CD3^+^CD4^+^ cells. Subpopulations of T lymphocytes CD4^+^ will be characterised as follows: Th1 as CD3^+^CD4^+^CD183^+^, Th2 as CD3^+^CD4^+^CD294^+^, Th17 as CD3^+^CD4^+^CD196^+^, Treg as CD3^+^CD25^+^CD127^-^ and neutrophils as CD15^+^CD11b^+^CD62L^+^/ CD15^+^CD11b^-^CD62L^+^ for “tethering” form, and CD15^+^CD11b^+^CD62L^-^ for active form. Due to high debris background in BALF samples, utilisation of the Viability dye is essential and utilisation of intracellular dyes is excluded. The data will be acquired using LSRII, BD Biosciences.

### 4.5 Inflammatory/antimicrobial markers and short chain fatty acids (SCFAs) analysis

Faecal samples will be analysed for calprotectin (kit Calprest NG, Eurospital, with an adaptation on BEP2000 (Siemens)), β-defensin 2 (β Defensin 2 ELISA Kit, Immundiagnostik, Bensheim), and HNP1-3 (human HNP1-3 ELISA Kit, Hycult biotech). All 3 markers will be measured in the Laboratory of Functional Coprologie, GH Pitié-Salpêtrière, Paris. The C-reactive protein will be measured in plasma by CRP human ELISA kit (Enzo Life Sciences). The SCFA concentration will be dosed after water extraction of acidified faecal samples using gas liquid chromatography (Nelson 1020, Perkin-Elmer) in the Commensals and Probiotics-Host Interactions Laboratory, Micalis Institute, INRA UMR 1319, France.

### 4.6 DNA extraction

A DNA extraction on all samples will be performed in batches to reduce the possibility of manipulation errors between extractions.

#### 4.6.1 Sample pre-treatment

**Lung tissue.** Lung tissue will be taken directly from liquid nitrogen, broken into smaller pieces with a mortar and pestle, and homogenised in Hank's balanced salt solution (HBSS) (Sigma-Aldrich) in gentleMACS M tubes (Miltenyi Biotec). The ratio of buffer volume:sample weight will be determined for each sample, and adapted volume will be used for each of the following steps. The programs used will be those adapted for lung and tumour tissue (Miltenyi Biotec). The homogenate obtained will be treated with collagenase D (Sigma-Aldrich) (2 mg/mL final concentration) at 37°C for 15 min, followed by 10 min at 2000 × g at room temperature (RT). The pellet will be resuspended in 2–5 mL (depending on the initial sample weight) of mammalian cell lysis buffer (MCLB),^[24]^ and repeatedly vortexed for 5 min at RT. The reaction will be stopped with adding an equal volume of neutralisation buffer.^[24]^ After 2 washes with PBS (Sigma) (2000 × g for 10 min), the pellet will be used for DNA extraction using the adapted protocol of Godon et al.^[25]^

**Saliva and BALF.** Saliva and BALF will first be brought to RT and vortexed. 1 mL of saliva and 5 mL of BALF will be used for DNA extraction. Whole BALF will be used for extraction, to minimise the loss of bacterial communities.^[26]^ BALF will be centrifuged (7000 × g, 10 min) and 3 mL of MCLB will be added to the pellet, while saliva will be treated directly with 1 mL of MCLB. Both will be vortexed for 5 min at RT, followed by addition of neutralisation buffer. DNA extraction will be performed directly on the pellet after centrifugation at 7000 × g for 10 min at RT and a washing step (PBS).

**Faeces.** Faecal samples will have no pre-treatment and extraction will begin directly on frozen samples.

#### 4.6.2 DNA extraction

DNA extraction will be performed by using the adapted protocol of Godon et al.,^[25]^ i.e. International Human Microbiome Standards Standard Operating Protocol for Fecal Samples (IHMS SOP) 07 V1. Briefly, 4 M guanidine thiocyanate and 10% N-lauroyl sarcosine will be added directly on frozen samples or pellets for 10 min at RT. After the addition of 5% N-lauroyl sarcosine and homogenisation by vortexing, the samples will be incubated for 1 h at 70°C. All of the samples will be transferred to Lysing Matrix B tubes (MPBio) and homogenised using FastPrep-24 Instrument (MPBio), 4 × 45 s at 6.5 ms^−1^. Between each cycle, the samples will be cooled on ice for 2 min. One micro-spoon of polyvinylpolypyrrolidone will be added to each tube, followed by vortexing and centrifugation for 3 min at 18,000 × g. The supernatant will be removed and placed in a new 2 mL tube and the pellet will be washed with TENP and centrifuged for 3 min at 18,000 × g, and the new supernatant will be added to that which was harvested previously. The pooled tube will be centrifuged for 1 min at 18,000 × g and the supernatant will be transferred to a new 2 mL tube. One volume of isopropanol will be added to the supernatant, gently mixed by turning the tube and incubated for 10 min at RT. After centrifugation for 5 min at 18,000 × g, the pellet will be resuspended in 0.1 M phosphate buffer, pH 8, and 5 M potassium acetate and incubated overnight at 4°C. The samples will then be centrifuged for 30 min at 18,000 × g and 4°C. The supernatant will be transferred to a new 2 mL tube, and after the addition of RNase (final concentration 40 μg/mL), incubated at 37°C for 30 min. Nucleic acids will be precipitated with absolute ethanol and 3 M sodium acetate, followed by centrifugation at maximum speed for 3 min. The pellet will be washed with 70% ethanol, dried and resuspended in 100 μL of TE buffer. DNA quality and concentration will be estimated by agarose gel electrophoresis and nanodrop (NanoDrop ND-1000) measurement, respectively.

Considering the low biomass samples, background controls have been made throughout the sampling and extraction process. The physiological serum used to perform BAL is 1st sampled with the same syringe that is afterward used for lavage from the same vessel containing physiological serum. This sample is used as a “negative sampling control”. During the DNA extraction, miliQ water is used as a “negative background control” sample, treated will all the reagents and passing all the procedures along with the real samples. These “negative” samples will be analysed along with the real samples.

All the reagents used in DNA extraction and sample pre-treatments were either autoclaved, filtered through 20 μm filters or purchased sterile. All the tools and pipettes were thoroughly washed and disinfected between extractions of different sample types, to minimise the transfer from high biomass samples. Also, DNA extraction from lung tissue samples (3 samples per patient) was randomised (each extraction “batch” never contained only one sample type from different patients or all the samples from the same patient), to minimise the “batch” effect.

### 4.7 Molecular analyses of microbiota

#### 4.7.1 The 16S rRNA gene sequencing

The genomic DNA from saliva, faeces, BALF, “healthy” lung, peritumoural and tumoural tissue, and negative controls will be analysed by sequencing of the bacterial 16S rRNA gene by DNAVision, Belgium, using Illumina MiSeq technology. After PCR amplification of the targeted region V3–V4, libraries will be indexed using the NEXTERA XT Index kit V2. The sequencing is carried out in paired-end sequencing (2 × 250 bp) by targeting an average of 10,000 reads per sample. Software used for bioinformatic analysis will be QIIME (Quantitative Insights Into Microbial Ecology), with a cut-off value of 5000 reads per sample for analysis. For each sample the following will be determined:

a) alpha and beta diversity,
b) comparison of alpha diversities based on a 2-sample *t* test using non-parametric (Monte Carlo) method,
c) statistical significance of sample groupings using distance matrices (Adonis method),
d) comparison of OTU frequencies across sample groups (comparison is performed at OTU, Phylum, Class, Order, Family and Genus level)

Multiple comparisons will be realised, both between different grouping criteria and between samples.

Considering the low taxonomic levels (Species), sequencing is best used as an indicative tool for further analyses by qPCR.

#### 4.7.2 qPCR

In our study, qPCR will be done in 2 phases:

a) Pre-16S sequencing analysis

In this phase, qPCR will have 2 purposes: 1st, to confirm and further characterise the bacterial functionary groups in faeces evaluated by bacterial culture (providing the information of viable bacteria inside specific functionary group); and 2nd, to quantify pathogens/commensals in respiratory and intestinal system, known to be implicated in tumourigenesis/pro or anti-inflammatory reactions^[27–32]^ using specific primers.

The qPCR will provide information of absolute quantity of taxa/species of interest in each sample, which is information that cannot be obtained by sequencing (only relative abundance).

b) Post-16S analysis

In our study, the sequencing has the purpose of sample “screening”. It will give us an idea of the composition of the microbial communities from different sites and originating from different conditions (patient with tumours eligible for chemotherapy/surgery) based on minimum of 5000 reads. However, sequencing stays a technique to determine relative abundance. Therefore, once the overall composition of each sample is determined, we will proceed with:

1. quantification of the specific outer taxonomic units (OTUs)/taxa we determine as relevant in either relative or normalised abundance not analysed during pre-16S analyses
2. enlarging the primer list specific for the new discovered OTUs of interest

The current list of primers optimised for our study is shown in Table 2. Each primer couple is tested for specificity on 60 referent species. The QPCR will be done using the Rotor-Gene Q machine (Qiagen). Additional primer couples will be tested and optimised if found necessary, as explained.

**Table 2:**
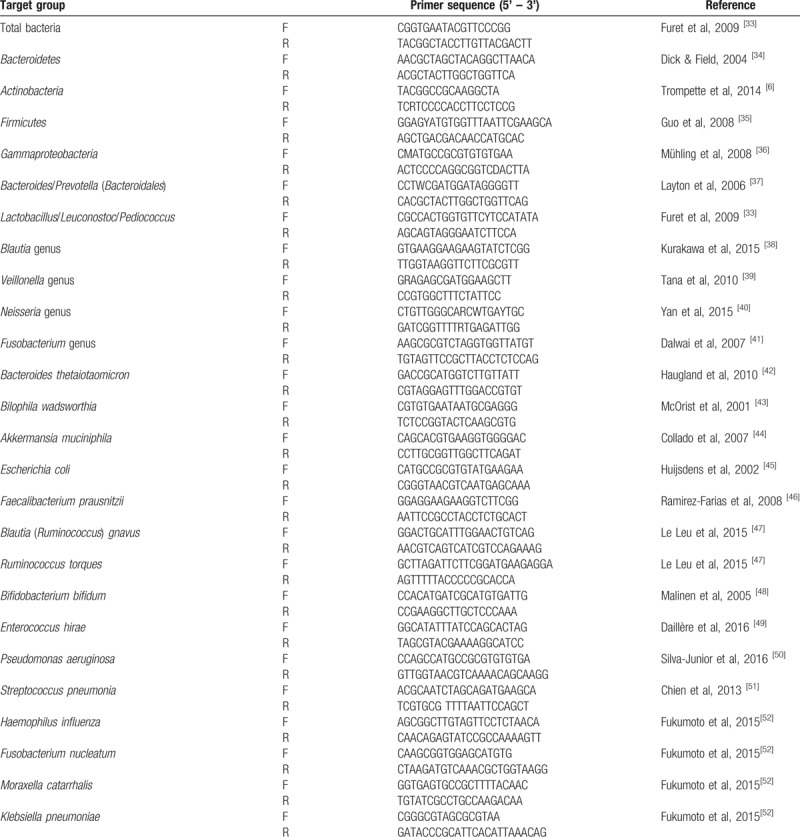
The current list of qPCR primer couples.

### 4.8 Statistical analysis plan

#### 4.8.1 General information

This study is exploratory and main outcomes address the description of microbiota characteristics in lung cancer patients. Three microbiota are concerned (gut, lung, and saliva) and data are collected at different times in patients treated by neoadjuvant chemotherapy (group Pct-chir). For other patients (group Pchir), different microbiota are sampled at only 1 time point, at surgery.

In patients treated by chemotherapy, analysis of variations of microbiota induced by chemotherapy will be possible by evaluation of changes in proportions over time. On the other hand, the analysis of microbiota with/without previous chemotherapy will only be descriptive, as the design is not compatible with a non-biased comparison of both groups.

#### 4.8.2 Sample size

The sample size calculation is based on the study of Montassier et al, 2015,^[53]^ where the abundance of the principal phylum *Firmicutes* in faecal microbiota decreased by approximately 30%, with an FDR-corrected *P*-value of .0002. Considering these results, 20 patients in the chemotherapy group should allow us to detect a similar variation in our samples, at least in the faecal microbiota. For group balance, the same sample size of 20 was retained for the surgery-only group.

#### 4.8.3 Description of patients’ characteristics

Patients’ characteristics will be described using standard distribution parameters: counts, range, mean/median, confidence intervals, standard deviation/interquartile range for quantitative parameters and, for categorical ones, counts and frequencies. This description will also be made by treatment group (Pchir/Pct-chir).

#### 4.8.4 Description of microbiota

Microbiota characteristics consist in several hierarchical steps including:

- Phylum: 4 main phyla are found in both lung and intestinal microbiota (*Firmicutes*, *Bacteroidetes, Actinobacteria*, and *Proteobacteria*). *Firmicutes* are supposed to represent ∼80% of the intestinal microbiota biomass^[54]^ and ∼40% of the lung microbiota.^[55]^ The proportion of each component will be described by its proportion of the biomass in %.
- Main classes of bacteria per phylum: these classes gather bacteria that share important characteristics and functions (e.g. *Bacilli*, *Clostridia*, *Gammaproteobacteria,* etc.). Composition of the microbial communities from different sites and originating from different conditions will be quantified (alpha and beta diversity, relative proportions).
- Inside classes, description by order, family and genus level will be performed when contributory on a biological plan.

Heterogeneity between microbiota will be studied. The comparison of proportions of phyla, or by other taxonomic level will be performed to evaluate if specific adaptation characterises the 3 microbiota and their components. The ANOVA will be used to perform inter-patient comparisons. The FDR correction will be applied when analyses are conducted within phyla.

#### 4.8.5 Comparison of microbiota before/after chemotherapy

This comparison will be performed for each site-specific microbiota. Proportions of main phyla will be compared using ANOVA (mixed model) to check if an independent chemo-effect can be objectivised, adjusting on patients and phyla (without FDR correction).

Comparisons of taxonomic levels below phylum before/after chemotherapy will be performed on relevant components with an FDR correction. These comparisons will be performed on both alpha and beta diversity. Univariate paired parametric or non-parametric tests (Student *t* test, Mann–Whitney *U* test, etc.) will be used here.

#### 4.8.6 Comparison of microbiota between the 2 treatment groups

These comparisons will use the same tests as in the previous paragraph, except tests will not be paired.

#### 4.8.7 Relationship between inflammatory status and microbiota

Several cytokines will be measured in blood samples. Each result will consist in a concentration of cytokine. The relationship between microbiota and inflammation will be tested using Pearson (or Spearman rank) correlation coefficient, using FDR correction.

#### 4.8.8 Tumoural immune infiltration

Immune reaction will be described by percentage by lymphocyte type. These proportions will be compared to corresponding microbiota characteristics: for example lung microbiota and lung tumour. Statistical association between these parameters will be tested as in the previous paragraph.

#### 4.8.9 Complementary analyses

Complementary analyses will be performed if particular biological issues can be better described.

All statistical analyses will be performed using R-software version 3.5.0 or later (R-Project, GNU GPL). Tests will be 2-sided and the significance threshold is set at 0.05, after FDR correction where needed (as for the analyses concerning taxonomic ranks below phylum). Data may be missing due to possible loss of follow-up between inclusion and end of study. A description of the missing data and associated reasons will be given.

### 4.9 Data monitoring committee

A data monitoring committee is not needed in this study since this is an observational trial and there are no intervention or security risks for patients.

## 5 Discussion

### 5.1 What is known

Lung cancer is a leading cause of death by cancer worldwide, responsible for 1,761,007 or 23.1% deaths in 2018 according to the WHO.^[56]^ It is also the most frequent cancer in men and the 3rd most frequent in women.^[56]^ While well characterised regarding its aetiology, morphological, and molecular properties,^[57–59]^ much less is known regarding its relationship with lung microbiota, and almost nothing regarding its connection to distant sites such as the gut and gut microbiota. This lack of studies is self-explanatory when one knows that not so long ago lungs were considered sterile except in case of infection.^[55,60]^ Recently, however, there is an emerging idea of more “systemic” influence of the gut microbiota, and its connection to the immune system beyond the local effect.^[3,60–62]^ A few teams made a huge leap in elucidating the role of the gut microbiota in chemotherapy and anticancer treatment, including lung cancer.^[63–67]^

### 5.2 What is new

Based on these studies and the questions unanswered, we designed a case-control observational trial underlining a multi-aspect approach to the patient. In each of our subjects, we decided to characterise the microbiota of different sites (UAs, gut, and lung microbiota), in parallel with immune profile characterisation (local and systemic) while taking into account the patient's life style (nutrition, smoking status, profession, etc.). Examination of these factors in patients undergoing chemotherapy before surgery enables a direct follow up of these parameters correlated with the treatment phase (to our knowledge, this has never been reported for lung cancer before). Moreover, lung microbiota at surgery is sampled in 2 ways: by performing broncho-alveolar lavage (BAL) directly on the excised lung lobe (to eliminate possible UAs contamination and obtain the maximal microbial concentration for analysis), and by sampling lung tissue at 3 sites: “healthy” tissue distal to tumour (used as a control tissue),^[12]^ peritumoural tissue, and tumour itself. This enables sampling of both luminal and tissue/cell-bound bacteria which, according to known studies, do not share the same microbial composition.^[26]^ To our knowledge, at present there is no study of lung cancer that examines the microbiota of peritumoural tissue, and even less in 4 different lung sample types. Also, no study performed BAL directly on the tumour lobe without passing through the UAs.

### 5.3 Choice of analyses

As previously reported, certain bacterial species can modify our immune responses differently, such as *Faecalibacterium prausnitzii* or on the other hand *Fusobacterium nucleatum*, as well as the whole cluster (*Clostridia* cluster XIV).^[68,69]^ Therefore, tumour lymphocyte infiltration and lymphocyte composition in the broncho-alveolar lavage fluid (BALF) will be examined and closely looked at for its relationship with sampled microbiota. Likewise, each tumour is characterised according to the TNM stage classification^[70]^ and histological properties. Tumour architecture, localisation in the lung, and disease severity are expected to dynamically interact with microbial composition in situ and immune profile, as seen in similar pathologic states of the lung (obstruction of the normal lung architecture, creation of anaerobic thermal pockets in the case of bronchial obstructiveness, immunogenicity of the tumour, promotion of neutrophil recruitment, inflammation).^[12,15,71–73]^ Difference of the microbiota composition between tumour samples of adenocarcinoma and squamous cell carcinoma has already been evidenced by Yu et al.^[15]^ Interestingly, salivary microbiota is also proven to correlate with NSCLC type.^[40]^ Therefore, similar analysis direction will be taken with our salivary and lung samples.

As mentioned, intestinal microbiota has both local and systemic influence on its host. According to the gut-lung axis theory,^[4]^ bacteria or their products might have systemic effects, and therefore, have an effect on the lung microbial composition and immune response. For this reason, faecal microbiota will be characterised, as well as faecal SCFAs concentrations (known products of bacterial fermentation and immune modulators/protectors of the intestinal barrier).^[74]^ The SCFAs might be potential mediators of the gut's “long-distance” influence by direct effect on the target site or indirectly via gut/circulating immune system stimulation. As intestinal microbiota is shown to adapt very quickly to the changes in nutrition, as well as its influence on SCFAs concentrations,^[22,75]^ nutritional records before each faecal sampling will be taken into account. We will not only determine the composition of faecal microbiota, but also its “quality” and influence on intestinal health by dosage of bacteriocins (HNP1-3, β-defensin 2), and calprotectin as inflammatory marker.^[76]^ Broad-spectrum cytokine profiling and immune cell phenotyping in the blood will be used to evaluate systemic immune status. This holds particular importance as connection to circulating IL-6 and IL-8 was previously reported in lung cancer,^[76,77]^ but also to intestinal SCFA concentrations.^[78]^

As explained, group Pct-chir will enable follow-up on multi-site microbiota and immune status during different treatment phases (Fig. 1). Since the biggest problem of chemotherapy, despite its efficacy against tumour cells, is its non-selectivity (effecting epithelial layers and mucosae),^[79–81]^ we expect to see changes in all 3 types of microbiota—salivary, faecal, and lung, as all are closely related to epithelial and mucosal layers. Similarly, immune characteristics should be altered following the chemotherapy and above-mentioned changes in microbiota (but also vice versa—the affected immune system will change its interaction with microbiota, thus modifying it). Finally, we could hypothesise that different initial properties of the tumour (why the patient is prescribed chemotherapy or not in the 1st place) might divide 2 patient profiles (Pchir vs. Pct-chir 1st time point) regarding both multi-site microbial and immune/inflammatory characteristics.

### 5.4 Final word

To conclude, our results will be one of the 1st to give a better understanding of the close and intense interaction between the microbiota of different, yet communicating sites and their interaction with the immune system in patients suffering from lung cancer (the world's number 1 cause of death by cancer).^[82]^ The strength of this study design is data collection through multiple non-invasive techniques that can be incorporated into the standard medical care and treatment of the patients. Another strong point is a multi-site approach towards each patient: lifestyle, nutrition, immune status, and microbial composition are assessed using different and complementary techniques (e.g. faecal microbiota will be assessed by techniques of molecular biology via qPCR and sequencing, but also by bacterial culture, and in the aspect of individual's nutrition). The main limitation is lack of the “healthy” control group, not authorised by Ethics Committee because of the invasiveness of the sampling techniques for healthy subjects. Therefore, previously published data on healthy subjects and similar cohorts will be addressed only in a descriptive matter, while we will focus more on relational aspects (e.g. interaction between site-specific immunity and its microbiota).

We hope that our results will help in setting the basis for developing more personalised or “alternative” approaches in lung cancer treatment, better characterisation of patient's status and diagnosis, as well as in finding ways of improving chemotherapy tolerance and effectiveness (complementary prebiotics, probiotics or symbiotics).

## Author contributions

The RB and EF wrote the manuscript. MF and EF proposed the study design and protocol preparation. JYB and ET were involved in the preparation of protocol amendments. MPV was involved in the study design. ABD and NRR led the study design and protocol preparation. FK wrote the statistical analysis plan. MF was responsible for patient recruitment, surgery procedure and lung sampling during surgery. Preparing the study design, sample and data collection, management, analyses and decision to submit the report for publication is the responsibility of RB, EF and MF. All authors have reviewed this manuscript.

**Conceptualization:** Marc Filaire, Nina Radosevic-Robin, Jean-Yves Berthon, Annick Bernalier-Donadille, Marie-Paule Vasson, Fabrice Kwiatkowski, Edith Filaire.

**Data curation:** Rea Bingula, Marc Filaire.

**Formal analysis:** Rea Bingula, Nina Radosevic-Robin.

**Funding acquisition:** Marc Filaire, Jean-Yves Berthon, Annick Bernalier-Donadille, Marie-Paule Vasson, Edith Filaire.

**Investigation:** Rea Bingula, Marc Filaire, Nina Radosevic-Robin.

**Methodology:** Rea Bingula, Marc Filaire, Nina Radosevic-Robin, Annick Bernalier-Donadille, Marie-Paule Vasson, Fabrice Kwiatkowski, Edith Filaire.

**Project administration:** Marc Filaire, Jean-Yves Berthon, Annick Bernalier-Donadille, Marie-Paule Vasson, Emilie Thivat, Edith Filaire.

**Software:** Fabrice Kwiatkowski.

**Supervision:** Marc Filaire, Nina Radosevic-Robin, Annick Bernalier-Donadille, Marie-Paule Vasson, Edith Filaire.

**Validation:** Marc Filaire, Nina Radosevic-Robin, Jean-Yves Berthon, Annick Bernalier-Donadille, Marie-Paule Vasson, Emilie Thivat, Fabrice Kwiatkowski, Edith Filaire.

**Visualization:** Rea Bingula.

**Writing – original draft:** Rea Bingula, Edith Filaire.

**Writing – review & editing:** Marc Filaire, Nina Radosevic-Robin, Jean-Yves Berthon, Annick Bernalier-Donadille, Marie-Paule Vasson, Emilie Thivat, Fabrice Kwiatkowski, Edith Filaire.

Rea Bingula orcid: 0000-0002-7782-7538.
